# Effectiveness of Behavioral Counseling in Smokeless Tobacco Cessation Among Adult Users Reporting to a Dental Hospital in Pune: A Randomized Controlled Trial

**DOI:** 10.7759/cureus.24041

**Published:** 2022-04-11

**Authors:** Abhishek Kumbhalwar, Sahana Hegde, Pradnya Kakodkar, Vini Mehta, Himanshu Gupte, Sudhir Jadhav

**Affiliations:** 1 Department of Public Health Dentistry, Dr. D. Y. Patil Dental School, Pune, IND; 2 Department of Public Health Dentistry, Dr. D. Y. Patil Dental College and Hospital, Dr. D. Y. Patil Vidyapeeth, Pune, IND; 3 Research, Dr. D. Y. Patil Vidyapeeth, Pune, IND; 4 Department of Public Health Dentistry, People's College of Dental Science and Research Center (PCDS), Bhopal, IND; 5 Department of Public Health Dentistry, Narotam Sekhsaria Foundation, Mumbai, IND; 6 Department of Community Medicine, Dr. D. Y. Patil Medical College, Hospital & Research Centre, Dr. D. Y. Patil Vidyapeeth, Pune, IND

**Keywords:** tobacco cessation, india, motivational interviewing, behavioral counseling, smokeless tobacco(st)

## Abstract

Aim

To assess the effectiveness of behavioral counseling for smokeless tobacco cessation among adult users in a dental hospital setting.

Methods

A total of 200 patients visiting a dental hospital who were exclusively using smokeless tobacco (SLT) were enrolled in the study. A randomized controlled trial with a concurrent parallel study design, which consisted of two arms, was conducted. Fagerstrom test for nicotine dependence level and the transtheoretical stage of change was assessed at the baseline. Behavioral cessation counseling and motivational interviewing were provided in the study arm and brief advice was given to those in the control arm. The counseling was provided at baseline and followed up till six months through telephone to assess the change in the frequency of use of SLT products and abstinence from SLT use. A biochemical validation with a urine cotinine test was done to confirm abstinence.

Results

At six months, there was a significant difference within and between the study and control groups, indicating the role played by behavioral tobacco cessation in reducing the frequency of consumption. About 24.4% of participants in the study group and 10% in the control group abstained from the habit at the sixth month, with an odd’s ratio (OR)=2.91 and with a loss to follow-up of 10% in each of the groups. The cotinine test, which was used for validation, revealed a significant difference between the study and the control group. The number needed to treat (NNT) shows that to motivate one additional person to give up the SLT habit, we need to intervene with about seven people.

Conclusion

Behavior intervention with motivational interviewing was considered an effective method in promoting smokeless tobacco cessation among adults. Transtheoretical stages of change have proven to be an effective model to assess the stage of behavior change of the population toward SLT use and was also helpful for changing the behavior.

## Introduction

Oral cancer is one of the most common cancers in India, which is partially attributable to using smokeless tobacco (SLT). According to the World Health Organization (WHO), tobacco-related fatalities in India might reach 1.5 million per year by 2020 [1]. Indian healthcare experts advised 32% of SLT users to quit smoking, while 49.6% of smokeless tobacco users were considering quitting [2,3]. According to a Cochrane evaluation published (2016), smokers who received text-message-based help were 1.7 times more likely to quit than smokers who did not, demonstrating the efficacy and utility of this intervention for SLT users [4]. According to MPOWER 2017, India's national, bilingual mCessation program (text-message-based help) started in 2016. After six months, an average quit rate of roughly 7% was observed among smokers and SLT users, according to an analysis of over 12,000 registered users at the conclusion of the first year [5].

Behavioral counseling can boost tobacco abstinence rates among SLT users regardless of whether they are seeking treatment or not. However, the methodological quality being limited mitigates the strength of this conclusion. Behavioral intervention with telephonic support counseling showed better results for quitting. Motivational interviewing (MI) is a form of counseling technique that has been shown to improve SLT users' cessation rates among those who have a low desire to quit and resist participation in cessation programs [6]. Its use by health experts has recently been discovered to be promising, as it is typically used by skilled cessation counselors in high-income countries [7,8].

A paucity of literature is evident about SLT cessation intervention trials internationally, with only 3% of parties (5/179 parties) reporting findings, i.e., United Kingdom (UK), Sweden, India, Norway, and Pakistan have published reports [9]. Evidence shows that a short dental setup-based intervention can be beneficial for SLT cessation [10]. Patients who visit the dental hospital may not be motivated to quit the habit of tobacco chewing. Tobacco smokers who receive intervention from oral health professionals in the dental office are more likely to quit [10]. Nevertheless, due to the small number of trials evaluated, it is difficult to establish the most important component of the cessation intervention [10]. Since using SLT can be harmful to health, an attempt was made to ask them to give up the habit through behavioral counseling and motivational interviewing, along with telephonic support with data fed using an electronic app [11]. Thus, the study was planned with the aim of assessing the effectiveness of behavioral counseling for smokeless tobacco cessation among adult users in a dental hospital setting.

## Materials and methods

Study setting and population

A randomized controlled trial with a concurrent parallel design was conducted on patients visiting a dental college for oral health check-ups and dental treatment. We included all participants over the age of 18 who had been using smokeless tobacco exclusively for at least one month prior to the visit. Participants with any acute or chronic disease that prevented them from effectively participating in the experiment, such as blindness, deafness, or intellectual handicap, were excluded from the study. Additionally, depending on his evaluation of the capacity to engage effectively in the trial and follow-up at the end of the intervention period, the principal investigator had the authority to exclude any eligible patient. Participants were informed of study details, including intervention, follow-ups, confidentiality, and the voluntary nature of participation, before signing the consent form. Walsh et al. [12] observed quit rates of 34.5% and 15.9% for the intervention and control groups for smokeless tobacco among college athletes, respectively. These proportions and the loss to follow-up were taken into account for sample size estimation. A total of 100 participants in each arm were recruited.

Ethics approval of research

Ethical clearance was obtained from the Institutional Review Board of Dr. D. Y. Patil Vidyapeeth, Pune with approval number DPU/R&R(D)/971(36)/16.

Randomization, allocation, and blinding

Participants were recruited by the principal investigator using a computer-based randomization system to allocate participants in a 1:1 ratio into either study or control groups by permuted blocks while maintaining concealment. An allocation sequence was generated before assigning the participants to a particular group. Participants as well as the investigator enrolling participants could not foresee the assignment, and a random list of numbers was generated. It was a double-blind study where participants and the statistician were blinded to the assignment to intervention.

Intervention

The Fagerstrom Test for Nicotine Dependence (FTND) for smokeless tobacco [13] and the transtheoretical model of behavior change [14] were assessed at baseline. The two main components of the intervention included behavioral counseling, which consisted of 5 A’s and 5 R’s [15] and MI [16]. LifeFirst foundation [11] trained the tobacco cessation counselor to stimulate, create capacity, and provide high-quality, proven tobacco treatment. Counselors went through a week-long didactic course with interactive role-playing to understand smokeless cessation behavioral therapy and motivational interviewing strategies. Additionally, the counselor had access to a picture panel containing short and long-term ill effects of SLT use. Commcare was an electronic application (e-app) developed by Dimagi Inc. (Cambridge, MA, USA) [13] and optimized for optimal data management, prompt follow-ups, and monitoring. The nicotine cessation service was used to collect demographic information and SLT usage. The app also helps to streamline the process of patient identification and provides timely follow-up to quit the habit.

A counselor provided behavioral counseling which consisted of 5 A’s and 5 R’s [15]. Support was provided to each participant at different stages of behavior change (pre-contemplation, contemplation, determination, action, relapse, maintenance). The intervention followed a dynamic process in which triggers for each participant were identified and ways to cope up with the relapse were suggested. Participants who were using smokeless tobacco products for bowel movements were advised to have lukewarm water or other alternatives in the morning. The counselor motivated the participants using elements of MI [16] and participants were asked to set a ‘quit date’. Behavioral counseling and MI focused on motivating and preparing the participant to quit SLT use. The intervention was provided for 15-20 minutes to each participant. After the quit date, the intervention was focused on avoiding relapse. Telephonic follow-up was done on the seventh day, 15th day, first month, second month, fourth month, and sixth month for the interventional group to assess the change in the frequency of use of smokeless tobacco products, slips, relapses, and abstinence.

Control group participants received brief advice regarding the benefits of smokeless tobacco cessation at the start of the intervention, which included ask, warn, advice, refer, do (AWARD) [17]. A toll-free National tobacco quitline number (1800-112-356) was provided to the participants. The brief advice was provided for five to seven minutes for each participant. Telephonic follow-up was done on the seventh day, 15th day, first month, second month, fourth month, and sixth month for the control group to assess the change in the frequency of use of smokeless tobacco products, abstinence, and relapse of participants from SLT use.

Follow-up assessment procedures

Phone calls to the participants of the intervention and control group were made three times during every follow-up. If the participant did not receive the call any of the times, at any point interval then it was considered as a loss to follow-up. If the participants did not answer the phone calls at the fourth- and sixth-month's ends in both groups (study and control), they were also considered lost to follow-up.

Outcome measures

Biochemical validation of abstinence for study and control groups using the urine cotinine test kit Nano-Check^TM^ DAT (Nano-Ditech Corporation, Cranbury, NJ, USA) was done at the end of six months for those who self-reported to have remained abstinent from SLT use. The detection limit concentration (cutoff) of the urine cotinine test is cotinine at 200 mg/ml. Abstinence within and between study and control groups at the six-month end was being compared. 

Statistical analysis

The data were entered and analyzed using the Statistical Package for Social Sciences (SPSS) for Windows 26.0 (SPSS, Inc., Chicago, IL, USA). Confidence intervals (CIs) were set at 95%, and a p-value ≤ of 0.05 was considered as statistically significant. Descriptive statistics were applied for demographic information. A Mann-Whitney U test was applied between the study and control groups at baseline and six months for SLT participants. A Wilcoxon signed-rank test was applied within the study and control groups at baseline and six months for SLT participants. Odd’s ratio (OR) and 95% CI were reported.

## Results

**Figure 1:**
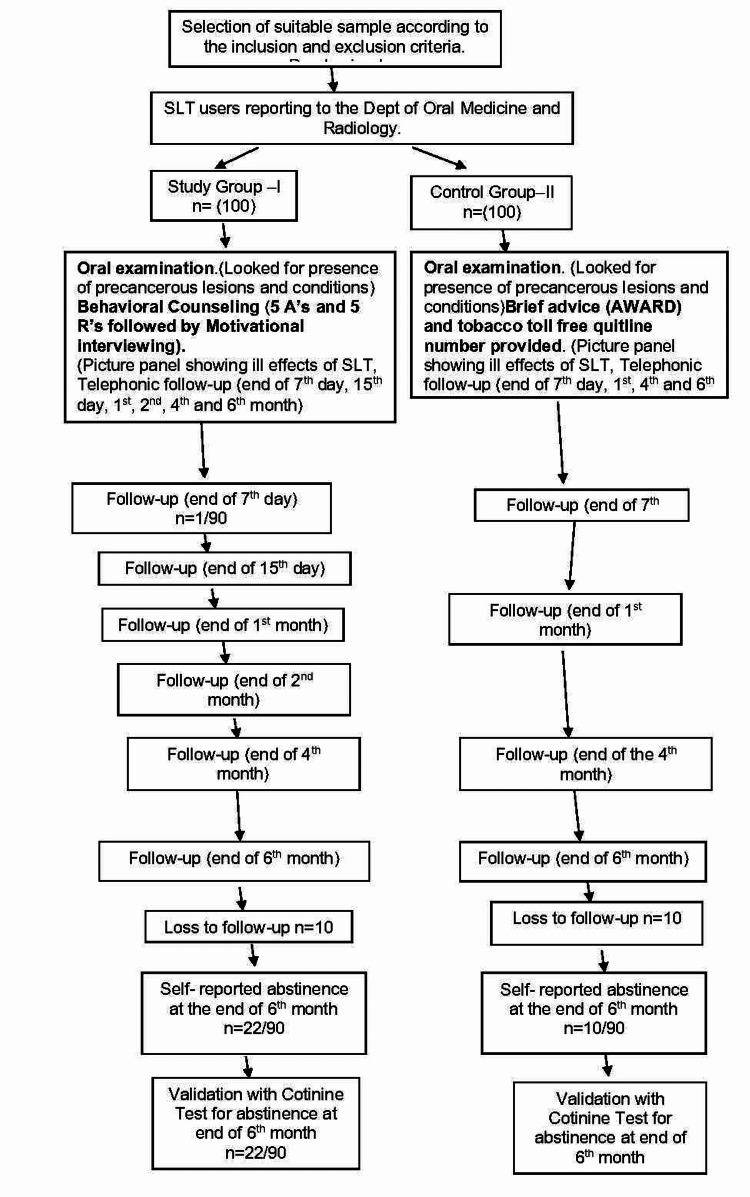
Patient flow from recruitment through sixth-month follow-up. SLT: smokeless tobacco, AWARD: ask, warn, advice, refer, and do.

The patient flow chart from recruitment to follow-up is shown in Figure 1. The current study presented 25 items of the electronic app (e-app) that were used for the collection of data [18]. 

Table 1 shows the demographic information of the study and control group participants. The values show that there is a statistical significance difference between the participants who abstained and non-abstained among the groups.

**Table 1:** Point prevalence of abstinence for smokeless tobacco.

	Study group	Control group	Total	p-value
Abstained	22 (24.4%)	9 (10%)	31	p=0.01
Not-abstained	68 (75.5%)	81 (90%)	149	
Total	90	90	180	

The groups were compared for the stages of change from baseline to the preparatory stage of the participants. The Chi-square test did not show a significant difference (p=0.69) between the groups at baseline for the transtheoretical stage of change and FTND score, indicating that a similar group entered the study (Table 2).

**Table 2:** Stages of change in baseline (N=200).

Stages of changes	Study group	Control group	Total	p-value
Pre-contemplation	61	61	122	p=0.69
Contemplation	35	37	72	
Preparation	4	2	6	
Total	100	100	200	

The behavioral control of the tobacco addicts was then compared among the groups so as to determine its influence on the subjects. SLT use at the baseline between the two groups was not significant, indicating baseline similarity between the groups. At six months, there was a significant difference within and between the study and control groups, indicating the role played by behavioral tobacco cessation in reducing the frequency of consumption (Table 3).

**Table 3:** Smokeless tobacco use among participants at baseline and sixth month.

	Baseline	Sixth month	Within the group
Study	4.67±3.00	2.63±2.66	Wilcoxon signed-rank test=8.14, p=0.001
Control	4.43±2.67	3.07±2.56	Wilcoxon signed-rank test=8.21, p=0.001
	Mann-Whitney U test t=0.64, p=0.52	Mann-Whitney U test t=2.15, p=0.03	

The demographic data of the patients were evaluated, and the table is illustrated below. About 24.4% of participants in the study group and 10% in the control group abstained from the habit at the sixth month, with an OR=2.91 and with a loss to follow-up of 10% in each of the groups. The cotinine test, which was used for validation revealed, a significant difference between the study and the control group. The number needed to treat (NNT) shows that to motivate one additional person to give up the SLT habit, we need to intervene with about seven people. Ten participants dropped out from the research (Table 4). 

**Table 4:** Demographic details of the participants (N=200). HSC: higher secondary certificate.

	Study group (%)	Control group (%)
Age group		
18-27	29	28
28-37	32	29
38-47	20	27
48-57	11	9
58-67	7	5
68-77	1	2
Mean±SD	36.6±8.38	27.25±11.35
Gender		
Male	88	83
Female	12	17
Literacy level		
Illiterate	5	2
No formal schooling but can read and write	1	1
Primary school completed (4th-7th std)	8	12
Secondary school/HSC completed (8th, 9th, 10th, 12th std completed)	57	60
College/university/postgraduate degree completed	29	25
Employment status		
Government/non-government/self-employed	73	68
Student/homemaker/retired	25	32
Unemployed, but able/unable to work	1	1

## Discussion

In India, among many causes, tobacco alone is found to be the most profound cause of avoidable morbidity and early mortality. From a public health standpoint, promoting cessation may stand a chance to be one of the most efficient preventive measures toward this avoidable morbidity and early mortality source. According to a review, varenicline, nicotine lozenges, and behavioral counseling may help SLT users discontinue. The studies included in this evaluation, however, were largely conducted in the United States and a few in Scandinavian countries (Sweden and Norway); South-East Asia Region (SEAR, India), Pakistan, and the United Kingdom were excluded [6].

World Health Organization 2017 reported that 20% of parties were providing tobacco cessation services at primary health centers, hospitals, offices of health professionals, or in similar setups. Most of these settings reported providing cessation support to smokers [19]. It was found in the present study that participants in the 28-37 year age group mostly used smokeless tobacco. Regarding literacy levels, for each participant, it was found that one-third of them had completed their higher secondary school. As shown by several global surveys [20], a strong predictor of tobacco use seems to be a lack of education. The same is re-iterated here. Almost three-fourths of the study population were self-employed. Ansari et al. discovered that tobacco use was highest in the labor class and among individuals with poor socioeconomic standing [21].

The majority of participants were in the pre-contemplation stage of change at baseline, followed by the contemplation stage. This was probably because the participants visited the hospital for oral treatment and not exclusively for tobacco cessation. However, there was no evidence that the effects were larger in a more motivated population [6]. Mushtaq et al. [22] reported that most of the participants who had registered for services with the Oklahoma Tobacco Helpline were in a contemplation phase like in this study. The current study found that most of the participants were in the low dependence group, followed by high dependence. Boyle et al. reported that low dependency has a more quitting success rate [23]. In the present study, 26% of low dependents from the study group had abstained from the habit at the end of the sixth month. Among a few tobacco chewers in this study, oral mucosal conditions like leukoplakia, oral submucous fibrosis (OSMF), erythroplakia, and tobacco pouch keratosis were noticed during an oral examination. Participants were made aware of the same during the oral examination and were briefed about the consequences. Smokeless tobacco users have high rates of mucosal pathology [24].

A consistent reduction in the frequency of use of SLT is being seen in both the study and control groups for the present study. A significant difference (p=0.03) in the reduction of SLT was seen among the study and control group participants at the sixth month, which indicates that the behavioral counseling through the A’s and R’s along with motivational interviewing indeed helped the participants. From baseline to sixth months, a significant difference (p=<0.01) was seen within the study and control groups, which indicates that the behavioral tobacco cessation and brief advice along with telephonic counseling helped the participants. Walsh et al. found that 34.5% of users from the intervention group and 15.9% from the control group quit their SLT use [12]. The intervention by the dental hygienist was marginally effective in reducing the usage of all tobacco products, particularly SLT use [25]. Similarly, counseling by a public health dentist helped the participants to give up the habit. In the present study, the tobacco products used by the participants were pan masala with tobacco, betel quid, gutkha, mishri, zarda (gai-chaap), tobacco with lime, and khaini. Though gutkha has been banned in Maharashtra, its use has been reported among participants as it is available in the black market [26].

In the study group, the seventh day of abstinence was 1.1%, whereas nobody gave up the habit among the control group in the present study. A randomized trial conducted by Virtanen et al. [27] used the 5 A’s approach and concluded that seven days of abstinence among snus users was 9.5% in the intervention and 4% in the control group. In the present study, fourth-month abstinence was seen among 12.2% of the study group and 3.3% of the control group participants. Behavior coping strategy, frequent phone contact, and awareness of health risks helped 36% of adolescent male users of SLT successfully quit [28]. It has been stated that free telephone support lines can provide cessation advice, quitlines, and counseling, which are predicted to enhance quit rates by roughly 4% [29]. The follow-up of participants using the telephone helped them. However, the percentage of those who abstained was lower compared to a few studies. Boyle et al. [23] reported a three-month point prevalence of abstinence to be 16.6% in manual only and 45.3% in telephone counseling groups. Masouredis et al. [30] showed a quit rate of 24% versus 15.9% when assessed at the end of three months. In their study, they considered behavioral counseling and follow-up telephone calls as the strongest tools for quitting of SLT.

In the present study, a self-reported abstinence of 24.4% and 11.1% was seen in the study group and control group, respectively. A post-biochemical validation of 24.4% abstinence among the study group and 10% abstinence among the control group was seen at the sixth month of follow-up. The statistical significant difference was seen between the two groups among the abstained (p<0.01). The success could be due to the reason that for the participants from the study group, the 5A’s and 5R’s and motivational interviewing were provided along with support according to the stages of change. Participants were advised to use the other substitutes like carom seeds, cardamom, and fennel seeds and were educated about coping with triggers like cravings, slips, and relapses and ways to take help from their family. Lichtenstein et al. [31] expressed the noteworthy role played by women through all stages of SLT cessation. The counselor expressed empathy and supported self-efficacy. Health education and counseling enabled women of Mumbai in low socioeconomic status (SES) to quit SLT at one-year follow-up with the quit rate of 33.5% [32]. Subjects in the behavioral intervention group were two times more prone to ceasing tobacco use compared to individuals in the simple advice group, where in 94% of SLT users were from lower SES strata [33]. About 12.5% had undetectable cotinine at six months when behavior change intervention for SLT cessation was supported [34]. In a systematic review and meta-analysis by Carr and Ebbert [10], brief advice from dental professionals was reported to be an efficient means to promote the stoppage of SLT use. Providing access to telephone support and oral examinations appeared to be beneficial in general, and quit rates for all tobacco use were lower than for SLT alone [6]. Boyle et al. [23] found that the six-month point prevalence of abstinence was 19.5% in the manual-only group and 42.3% in the telephone counseling group. At the conclusion of the 12th month, Walsh et al. [35] found a 27% vs 14% prevalence of self-reported cessation among athletes. Mushtaq et al. [22] reported that behavioral intervention was effective in ceasing tobacco use among SLT users. At one year, cessation was relatively high in both groups (36%) [36].

A urine cotinine test kit was used after urine samples were collected from participants who self-reported SLT abstinence. The sample was collected without any prior notice to the participants, though they were informed during the start of the study that urine may be collected at the end of the study period. At the end of six months, 22 participants in the study group and nine participants in the control group remained abstained. The urine cotinine test is considered as the biomarker in tobacco users, and the collection is non-invasive [37].

In both groups, there was a 10% loss to follow-up. Participants from the control group were provided with brief advice and a tobacco toll-free quitline number. This seemed to have helped the participants for quitting SLT. Mushtaq et al. [22] reported that 43% of tobacco users showed 30-day abstinence by using a telephone quitline number. In their study, they proved that an effective intervention to increase smokeless tobacco abstinence was tobacco quitlines. According to WHO 2017, 31% of parties have the provision of national quitlines (NQL) when compared among high and low socio-economic parties. It has been seen that 39% of high resource parties had NQL when compared to 21% of low socio-economic parties [9]. A quitline for all parties may be helpful.

Thus, global data revealed that a paucity of knowledge and adequate guidance for medical, dental, pharmacy, and nursing students in tobacco cessation existed among the majority of health care providers and school teachers [38]. With the aim of increasing awareness toward tobacco cessation and to know its importance, in 2018, the Dental Council of India has sent a notification to start a tobacco cessation center at every dental hospital, which means a total of 313 registered colleges will open up tobacco cessation centers, which will be a big step toward curbing the tobacco menace [39]. This is a worthwhile cause, and interns can be trained to counsel their patients during their first year of rotating internship, including post-graduate students and staff members. They can support their patients who use tobacco to give up the habit.

Strengths and limitations

This interventional study is the first of its kind at an Indian university, and it produced significant results that were validated by the cotinine test. Most of the patients want to discontinue but are ignorant about taking action. A public health dentist, counseling SLT users whom they come across routinely to give up, was a worthy cause. Patients visiting the dental college settings have high regard toward the counselor and are more inclined toward quitting the habit. Even a general dentist can counsel as he is treating patients for dental problems.

## Conclusions

Behavior intervention with motivational interviewing was found to be an effective strategy for encouraging adults to discontinue using smokeless tobacco. An oral examination of the patients who mostly belonged to the lower middle class by a dental professional could have played a role in promoting tobacco cessation. Transtheoretical stages of change have proven to be an effective model to assess the stage of behavior change of the population regarding smokeless tobacco use and was also helpful for changing behavior. Phone support also seemed to help. The low-cost efficacious public health interventions such as behavior intervention for cessation techniques have particular importance in developing nations where most of the burden lies and may gain even more importance while dealing with smokeless tobacco users.
